# Prevalence of Large‐for‐Gestational Age and Macrosomia Among Livebirths in 23 Low‐ and Middle‐Income Countries Between 2000 and 2021: An Individual Participant Data Analysis

**DOI:** 10.1111/1471-0528.70044

**Published:** 2025-11-10

**Authors:** Fati Kirakoya‐Samadoulougou, Joyeuse Ukwishaka, Calypse Ngwasiri, Seema Subedi, Elizabeth A. Hazel, Daniel J. Erchick, Lee Shu Fune Wu, Carlos Grandi, Carl Lachat, Laéticia Céline Toe, Dominique Roberfroid, Lieven Huybregts, Alain B. Labrique, Mabhubur Rashid, Saijuddin Shaikh, Rezwanul Haque, Abdullah H. Baqui, Samir K. Saha, Rasheda Khanam, Sayedur Rahman, Md Mariângela F. Silveira, Romina Buffarini, Roger L. Shapiro, Rebecca Zash, Zhonghai Zhu, Lingxia Zeng, Xiu Qiu, Jianrong He, Seifu H. Gebreyesus, Kokeb Tesfamariam, Grace Chan, Delayehu Bekele, Seth Adu‐Afarwuah, Kathryn G. Dewey, Stephaney Gyaase, Blair J. Wylie, Sunita Taneja, Ranadip Chowdhury, Giridhara R. Babu, R. Deepa, Mohamed Rishard, Marzia Lazzerini, Maria J. Rodriguez‐Sibaja, Sandra Acevedo‐Gallegos, Per Ashorn, Kenneth Maleta, Luke C. Mullany, Fyezah Jehan, Muhammad Ilyas, Stephen J. Rogerson, Holger W. Unger, Rakesh Ghosh, Sabine Musange, Aroonsri Mongkolchati, Paniya Keentupthai, Wafaie Fawzi, Dongqing Wang, Christentze Schmiegelow, Daniel Minja, Line Hjort, John P. A. Lusingu, Emily R. Smith, Honorati Masanja, Fathma Mohamed Kabole, Salim Nassir Slim, Abel Kakuru, Richard Kajubi, Dilys Walker, Peter Waiswa, Vundli Ramokolo, Wanga Zembe‐Mkabile, Katherine Semrau, Davidson H. Hamer, R. Matthew Chico, Enesia Banda Chaponda, Albert Manasyan, Jake M. Pry, Kebby Musokotwane, Bowen Banda, Andrew Prendergast, Bernard Chasekwa, Joanne Katz, Anne C. C. Lee, Robert E. Black, Subnational Collaborative Group for Vulnerable Newborn Prevalence, Hasmot Ali, Parul Christian, Rolf D. W. Klemm, Alan B. Massie, Maithili Mitra, Sucheta Mehra, Kerry J. Schulze, Abu Ahmed Shamim, Alfred Sommer, Barkat Ullah, Keith P. West, Nazma Begum, Nabidul Haque Chowdhury, Md. Shafiqul Islam, Dipak Kumar Mitra, Abdul Quaiyum, Modiegi Diseko, Joseph Makhema, Yue Cheng, Yixin Guo, Shanshan Yuan, Meselech Roro, Bilal Shikur, Yahya Mohammed, Sebastien Haneuse, Bezawit Hunegnaw, Yunhee Kang, Alemayehu Worku, Seyram Kaali, Charles D. Arnold, Darby Jack, Seeba Amenga‐Etego, Lisa Hurt, Caitlin Shannon, Seyi Soremekun, James M. Tielsch, R. D. Thulasiraj, Nita Bhandari, Jose Martines, Sarmila Mazumder, Yamuna Ana, Lotta Hallamaa, Juha Pyykkö, Mario I. Lumbreras‐Marquez, Claudia E. Mendoza‐Carrera, James M. Tielsch, Subarna K. Khatry, James M. Tielsch, Subarna K. Khatry, Atiya Hussain, Muhammad Karim, Farzana Kausar, Usma Mehmood, Naila Nadeem, Muhammad Imran Nisar, Muhammad Sajid, Ivo Mueller, Maria Ome‐Kaius, Elizabeth Butrick, Felix Sayinzoga, Ilaria Mariani, Willy Urassa, Thor Theander, Phillippe Deloron, Birgitte Bruun Nielsen, Alfa Muhihi, Ramadhani Abdallah Noor, Ib Bygbjerg, Sofie Lykke Moeller, Fahad Aftab, Said M. Ali, Pratibha Dhingra, Usha Dhingra, Arup Dutta, Sunil Sazawal, Atifa Suleiman, Mohammed Mohammed, Saikat Deb, Moses R. Kamya, Miriam Nakalembe, Jude Mulowooz, Nicole Santos, Godfrey Biemba, Julie M. Herlihy, Reuben K. Mbewe, Fern Mweene Hamomba, Kojo Yeboah‐Antwi, Jane Bruce, Daniel Chandramohan

**Affiliations:** ^1^ Department of International Health Johns Hopkins Bloomberg School of Public Health Baltimore Maryland USA; ^2^ Centre de Recheche en Epidemiologie, Biostatistique et Recherche Clinique, Ecole de Santé Publique Université Libre de Bruxelles Brussels Belgium; ^3^ Argentine Society of Paediatrics, Ciudad Autónoma de Buenos Aires Buenos Aires Argentina; ^4^ Department of Food Technology, Safety and Health Ghent University Ghent Belgium; ^5^ Department of Medicine Namur University Namur Belgium; ^6^ Nutrition, Diets, and Health Unit International Food Policy Institute Washington District of Colombia USA; ^7^ Noora Health Dhaka Bangladesh; ^8^ Centre for Health Research and Development, Society for Applied Studies Dehli India; ^9^ JiVitA Maternal and Child Health Research Project Rangpur Bangladesh; ^10^ Child Health Research Foundation Dhaka Bangladesh; ^11^ Projahnmo Research Foundation Dhaka Bangladesh; ^12^ Postgraduate Program in Epidemiology Federal University of Pelotas Pelotas Brazil; ^13^ Department of Pediatrics, Children's Hospital of Philadelphia University of Pennsylvania Philadelphia Pennsylvania USA; ^14^ Beth Israel Deaconess Medical Center Boston Massachusetts USA; ^15^ Department of Epidemiology and Biostatistics, School of Public Health Xi'an Jiaotong University Health Science Center Xi'an China; ^16^ Guangzhou Women and Children's Medical Center Guangzhou Medical University Guangzhou China; ^17^ Department of Nutrition and Dietetics, School of Public Health Addis Ababa University Addis Ababa Ethiopia; ^18^ Department of Public Health, School of Medicine and Health Sciences Ambo University Ambo Ethiopia; ^19^ Department of Obstetrics and Gynecology St. Paul's Hospital Millennium Medical College Addis Ababa Ethiopia; ^20^ Department of Nutrition and Food Science University of Ghana Accra Ghana; ^21^ Institute for Global Nutrition, Department of Nutrition University of California Davis California USA; ^22^ Biostatistics Unit Kintampo Health Research Centre Kintampo Ghana; ^23^ Division of Maternal‐Fetal Medicine, Department of Obstetrics and Gynecology Columbia University Medical Center New York New York USA; ^24^ Centre for Health Research and Development, Society for Applied Studies New Delhi India; ^25^ Department of Population Medicine, College of Medicine, QU Health Qatar University Doha Qatar; ^26^ PHFI Center for Developmental and Lifecourse Research Bengaluru India; ^27^ Faculty of Medicine, Department of Obstetrics & Gynaecology University of Colombo Colombo Sri Lanka; ^28^ Institute for Maternal and Child Health—IRCCS “Burlo Garofolo” WHO Collaborating Centre for Maternal and Child Health Trieste Italy; ^29^ Department Infectious Diseases Epidemiology and International Health London School of Hygiene & Tropical Medicine London UK; ^30^ National Institute of Perinatology, Maternal‐Fetal Medicine Department Mexico City Mexico; ^31^ Center for Child, Adolescent and Maternal Health Research, Faculty of Medicine and Health Technology Tampere University and Tampere University Hospital Tampere Finland; ^32^ College of Medicine University of Malawi Zomba Malawi; ^33^ Department of Paediatrcis and Child Health The Aga Khan University Karachi Pakistan; ^34^ The Aga Khan University Karachi Sindh Pakistan; ^35^ Department of Infectious Diseases, Doherty Institute University of Melbourne Melbourne Australia; ^36^ Menzies School of Health Research Darwin Australia; ^37^ Institute for Global Health Sciences University of California San Francisco San Francisco California USA; ^38^ School of Public Health, College of Medicine and Health Sciences University of Rwanda Butare Rwanda; ^39^ ASEAN Institute for Health Development Mahidol University Salaya Nakorn Pathom Thailand; ^40^ College of Medicine and Public Health Ubon Ratchathani University Ubon Ratchathani Thailand; ^41^ Harvard TH Chan School of Public Health Boston Massachusetts USA; ^42^ Department of Global and Community Health, College of Public Health George Mason University Fairfax Virginia USA; ^43^ Department of Immunology and Microbiology, Centre for Translational Medicine and Parasitology University of Copenhagen Copenhagen Denmark; ^44^ Department of Infectious Diseases Copenhagen University Hospital Copenhagen Denmark; ^45^ Department of Gynaecology and Obstetrics Copenhagen University Hospital–North Zealand Hilleroed Denmark; ^46^ National Institute for Medical Research Dar es Salaam Tanzania; ^47^ Faculty of Health and Medical Sciences, Novo Nordisk Foundation Center for Basic Metabolic Research University of Copenhagen Copenhagen Denmark; ^48^ Department of Obstetrics, Center for Pregnant Women with Diabetes Copenhagen University Hospital Copenhagen Denmark; ^49^ Department of Global Health George Washington University Milken Institute School of Public Health Washington District of Columbia USA; ^50^ Ifakara Health Institute Dar es Salaam Tanzania; ^51^ Ministry of Health Zanzibar Tanzania; ^52^ Infectious Diseases Research Collaboration Kampala Uganda; ^53^ Institute for Global Health Sciences and Bixby Center for Global Reproductive Health University of California San Francisco San Francisco California USA; ^54^ Department of Health Policy Planning and Management Makerere University School of Public Health, New Mulago Hospital Complex Kampala Uganda; ^55^ Division of Global Health, Department of Public Health Sciences Karolinska Institute Stockholm Sweden; ^56^ HIV Prevention Research Unit South African Medical Research Council Cape Town South Africa; ^57^ Health Systems Research Unit South African Medical Research Council Cape Town South Africa; ^58^ Ariadne Labs Brigham and Women's Hospital and Harvard TH Chan School of Public Health Boston Massachusetts USA; ^59^ Division of Global Health Equity & Department of Medicine Brigham and Women's Hospital Boston Massachusetts USA; ^60^ Department of Global Health Boston University School of Public Health Boston Massachusetts USA; ^61^ Section of Infectious Diseases, Department of Medicine Boston University Chobanian and Avedisian School of Medicine Boston Massachusetts USA; ^62^ Department of Disease Control, Faculty of Infectious & Tropical Diseases London School of Hygiene & Tropical Medicine London UK; ^63^ Department of Biological Sciences, School of Natural Sciences University of Zambia Lusaka Zambia; ^64^ Department of Pediatrics University of Alabama at Birmingham Birmingham Alabama USA; ^65^ Division of Epidemiology, School of Medicine University of California Davis California USA; ^66^ National HIV/AIDS/STI/TB Council of Zambia Lusaka Zambia; ^67^ Research Unit for Environmental Sciences and Management North‐West University Potchefstroom South Africa; ^68^ Queen Mary University of London London UK; ^69^ Zvitambo Institute for Maternal and Child Health Research Harere Zimbabwe; ^70^ Warren Alpert Medical School Brown University Providence Rhode Island USA

**Keywords:** birth weight, large‐for‐gestational age, low‐ and middle‐income countries, macrosomia, vulnerable newborn types

## Abstract

**Objective:**

To examine the prevalence of large‐for‐gestational age (LGA) and macrosomia in 23 countries between 2000 and 2021.

**Design:**

Descriptive multi‐country secondary data analysis.

**Setting:**

Subnational, population‐based cohort studies (*k* = 45 for LGA, *k* = 25 for macrosomia) in 23 low‐ and middle‐income countries (LMICs).

**Population:**

Liveborn infants.

**Methods:**

We conducted a secondary analysis of individual‐level data from the Vulnerable Newborn Measurement Collaboration, using INTERGROWTH‐21st standards to define LGA (> 90th centile for gestational age and sex) and macrosomia (≥ 4000 g, regardless of gestational age). We included LMIC population‐based datasets with reliable gestational age and birthweight data, excluding studies with small sample sizes, high missing data, or implausible measurements. Prevalence estimates were stratified by region, study period and gestational age, and results were summarised as medians and interquartile ranges (IQR).

**Main Outcome Measures:**

Prevalence of LGA and macrosomia.

**Results:**

Among 476 939 live births, the median prevalence of LGA was 5.1% (IQR: 2.9%–9.6%) and was highest in Latin America and the Caribbean at 9.6% (4 studies, IQR: 2.7%–16.1%) and lowest in South Asia at 2.7% (13 studies, IQR: 2.3%–3.7%). Over time, the median LGA prevalence increased from 4.9% (12 studies; IQR: 4.1%–7.9%) during the period from 2000 to 2010 to 5.9% (33 studies, IQR: 2.7%–11.2%) from 2011 to 2021. Term LGA was more common at 3.2% (0.9–5.1) than preterm or post‐term LGA. Among 313 064 live births, the median prevalence of macrosomia was 1.3% (*n* = 313 064, IQR: 0.2%–2.4%), which was highest in Latin America and the Caribbean (4 studies, 3.1%, IQR: 0.7%–6.8%) and lowest in South Asia (8 studies, 0.1%, IQR: 0.0%–0.7%). The median prevalence remained stable over time: 1.1% (8 studies, IQR: 0.2%–3.1%) in older studies (2000–2010) and 1.3% (17 studies, IQR: 0.5%–2.4%) in more recent studies (2011–2021). Term macrosomia was more common at 1.2% (0.2–2.0) than preterm and post‐term macrosomia.

**Conclusions:**

The overall prevalence of LGA and macrosomia was lower in these LMIC studies than is reported in high‐income countries. The prevalence of large babies was highest in Latin America and the Caribbean.

## 1 Introduction

Globally, the consequences of being born large‐in‐size have received comparatively less attention than babies born small, despite both conditions being at increased risk for neonatal morbidity, mortality and long‐term health complications. Hence, global monitoring of the prevalence, outcomes and life trajectory of large‐for‐gestational‐age (LGA; > 90th centile for gestational age and sex) and macrosomic (≥ 4000 g) infants is crucial due to their elevated morbidity and mortality compared to peers born appropriate‐for‐gestational‐age [1, 2, 3]. Infants with LGA or macrosomia have increased risk of acute perinatal complications including shoulder dystocia, birth asphyxia, prolonged hospitalisation, hypoglycaemia, respiratory diseases and death [4, 5, 6]. In later life, they are at higher risk of developing obesity, psychiatric disorders, cardiometabolic diseases and cancer during childhood or adulthood [4, 7, 8, 9, 10, 11, 12, 13].

Macrosomia is less sensitive in identifying at‐risk infants than LGA, which considers both sex and gestational age (GA), although its sensitivity can also depend on the choice of the growth chart used and accuracy of GA measurement [10]. Researchers often classify macrosomia into three grades: Grade 1 (4000–4499 g), Grade 2 (4500–4999 g) and Grade 3 (≥ 5000 g) [14]. The literature indicates that a newborn weighing ≥ 4000 g at birth is above the 90th percentile of weight relative to GA [15]. However, in some countries with population genetics and lifestyles that are associated with larger birth weights, stricter definitions are used. In these settings, LGA is defined as a birth weight > 97th percentile, and macrosomia as a birth weight ≥ 4500 g, allowing for more precise identification of infants at the highest risk of perinatal morbidity and mortality [14, 16, 17, 18]. The aetiology of large size at birth may be due to excessive foetal weight gain during pregnancy or a prolonged gestation period, both influenced by genetic and maternal factors as well as health system strategies for managing late‐term and post‐term births. LGA includes subgroups of constitutionally large and overweight babies, each facing distinct risks of clinical complications [19].

Considerable amounts of evidence show a rising prevalence of LGA and macrosomia, linked to the increasing prevalence of gestational diabetes and obesity in pregnancy [20, 21, 22, 23]. However, systematic global or regional estimates of LGA, along with time‐series analyses, have been largely limited to high‐income countries. Studies using the standardised INTERGROWTH‐21st criteria have demonstrated varying prevalence across different populations, ranging from 8.0% to 25.1% in Australia and across 16 European cohorts [24, 25, 26, 27]. A recent analysis of 115 million live births from 15 middle‐ and high‐income countries revealed that about 20% of all live births were classified as LGA and 10% were considered macrosomic. However, this did not indicate increased vulnerability for newborns partly due to the use of the 90th percentile and birth weight threshold of 4000 g rather than the stricter definitions [2]. In contrast, in low‐ and middle‐income countries (LMICs) where the burden of maternal and neonatal morbidity and mortality is greater, research on the impact of being born LGA is limited in scope, and global estimates of LGA remain unavailable to date. Previous reports of the prevalence of macrosomia in 23 LMICs showed varying results, ranging from 1% to 14.9% in 2013 [28]. However, updated reports are lacking in LMICs.

To address this gap in knowledge, we analysed data from 23 LMICs to describe the prevalence of LGA and macrosomia among live births. This research is part of a series aimed at enhancing the evaluation and measurement of newborn vulnerability via a classification system called vulnerable newborn types that combines gestational age (term [T] vs. preterm [PT]) and newborn size (small for gestational age [SGA]); appropriate for gestational age (AGA); or LGA; and uses the INTERGROWTH 21st international standards as the reference population [1, 2].

## 2 Methodology

### 2.1 Study Design and Data Source

This study is a descriptive, individual participant secondary data analysis examining the prevalence of LGA and macrosomia using data sourced from subnational population‐based studies conducted in LMICs. We identified relevant datasets containing information on GA, sex and birthweight of newborns through a comprehensive search of published literature, professional networks, online database searches and collaboration with investigators from LMICs (Figure S1). Studies resulting from the search terms used were retrieved from databases including MEDLINE, Embase, Scopus and OVID Global Health, covering articles published between 1 January 2000 and 15 March 2021. These datasets were compiled for the Vulnerable Newborn Measurement Collaboration and included data from 23 countries: Argentina, Brazil, Guatemala and Mexico in Latin America and the Caribbean; Botswana, Burkina Faso, Ethiopia, Ghana, Malawi, Rwanda, South Africa, Tanzania, Uganda, Zambia and Zimbabwe in sub‐Saharan Africa; Bangladesh, India, Nepal, Pakistan and Sri Lanka in South Asia; and China, Papua New Guinea and Thailand in Eastern and South‐Eastern Asia and Oceania. These datasets were compiled for the Vulnerable Newborn Measurement Collaboration. Further methodological details regarding study identification are available elsewhere [29].

### 2.2 Variables

The primary outcome of interest was LGA, defined as a birth weight greater than the 90th percentile for gestational age and sex, based on the INTERGROWTH‐21st international newborn size standards. In studies with available original data, macrosomia was the secondary outcome. We considered macrosomia first, then a three‐grade system (Grade 1: birthweight ≥ 4000 g, Grade 2: 4500–4999 g and Grade 3: ≥ 5000 g) [14]. For this study, we specifically focused on birthweight data collected within 72 h of delivery. The factors considered in this study included the length of pregnancy, method of GA assessment (ultrasound [U/S], last menstrual period [LMP] or both), region of the study country, the study setting (rural, urban or mixed) and period (older studies conducted between 2000 and 2010 or more recent studies between 2011 and 2021). The studies used a mix of GA assessment methods, primarily based on LMP and/or ultrasound, where available. Early ultrasound dating (< 14 weeks gestation), considered the gold standard, was not consistently implemented across studies. When ultrasound data were available, the earliest scan was typically used for GA estimation. Discrepancies between LMP and ultrasound estimates were resolved according to the protocol of each original study, which varied. Some studies applied standard clinical algorithms to select the most reliable GA estimate, while others relied solely on LMP [29].

### 2.3 Inclusion and Exclusion Criteria and Assessment of Data Quality

The study included datasets compiled for the Vulnerable Newborn Measurement Collaboration, specifically from LMICs. We excluded studies that did not meet pre‐defined population‐based criteria for participant recruitment. Facility‐based studies were considered population‐based if about 80% or more of births occurred in facilities. Studies that recruited participants from antenatal clinics (ANC) were considered population‐based if around 90% of women had at least one ANC visit in the sampled area. Furthermore, we also excluded studies that had fewer than 300 live births, did not utilise U/S or LMP for GA assessment, or failed to collect birthweights within 72 h of delivery. Additionally, studies with more than 30% missing data on GA, birthweight and sex were excluded, along with those where birthweights were improbable (< 250 g or ≥ 6500 g) or where GA fell outside the range of 22–44 weeks because the INTERGROWTH 21st reference does not have weights for these GAs. Furthermore, combinations of GA and birthweight that exceeded five standard deviations from the mean weight for each week of gestation, stratified by sex, were deemed improbable and excluded.

During the initial data cleaning process, standardised coding was used by all authors to ensure uniform inclusion and exclusion criteria across all datasets. We retained multiple births in this analysis because we aimed to describe the prevalence of large birth types among live births. Among randomised controlled trials included in this analysis, all participants were retained, regardless of their intervention assignment [29].

### 2.4 Statistical Analysis

A descriptive analysis was conducted to examine the prevalence of LGA in 45 studies from 23 LMICs. Stratified analyses were performed by region, study period, setting and GA measurement method to describe variations in LGA prevalence. The analysis also included a subsample of LGA infants to determine the proportions of preterm (< 37^+0^ weeks gestational age), term (37^+0^ to 41^+6^ weeks gestational age) and post‐term (≥ 42^+0^ weeks gestational age). For a subset of 25 studies from 19 countries for which original birth weight data were provided by authors, we also calculated the prevalence of macrosomia. The calculated prevalences were presented as medians with interquartile ranges (IQR) since they are not representative of the entire region or country because they represent sub‐national studies rather than nationally representative sampling.

### 2.5 Ethical Consideration

The Vulnerable Newborn Measurement Collaboration received ethical clearance from the Institutional Review Boards of the London School of Hygiene & Tropical Medicine (reference: 22858, approval date: 17 May 2021) and the John Hopkins Bloomberg School of Public Health (IRB No: 16439, approval date: 8 May 2021). Ethical approvals for the primary studies were obtained from all 23 countries' teams from their respective ethical bodies.

## 3 Results

For LGA prevalence, we included 45 studies from 23 countries, comprising 476 939 live births. These studies spanned 11 countries in sub‐Saharan Africa, 5 in South Asia, 3 in East Asia, South‐East Asia and Oceania, and 4 in Latin America and the Caribbean. Most of the data came from sub‐Saharan Africa (53.3%, 24 studies, 247 040 live births), followed by South Asia with 28.9% (13 studies, 166 149 live births). The study publication periods ranged from 2000 to 2010 (12 studies, 85 052 live births) and from 2011 to 2021 (33 studies, 391 887 live births) (Table S1).

In the 23 LMICs across 45 studies and 476 939 live births, a total of 41 797 live births were classified as LGA. The overall median prevalence was 5.1% (IQR: 2.9%–9.6%) (Table 1). The median prevalence was highest in studies from Latin America and the Caribbean at 9.6% (IQR: 2.7%–16.1%), followed by sub‐Saharan Africa at 8.5% (4.2%–12.7%) and the Eastern, Southeastern, and Oceania regions at 7.9% (6.7%–8.8%). The lowest median prevalence was observed in studies from South Asia (2.7%, IQR: 2.3%–3.7%) (Table 1). The highest median prevalence of term LGA was most common in Latin America and the Caribbean (7.3%, IQR: 2.3–13.9) and the highest preterm LGA was reported in sub‐Saharan Africa (2.9%, IQR: 0.7–6.9) (Table 2).

**TABLE 1:** Median prevalence of large for gestational age infants in 45 studies in low‐ and middle‐income countries.

	Total studies	Total live births	Median prevalence (IQR)
Total	*k* = 45 studies	*n* = 476 939	5.1 (2.9–9.6)
*By region*			
Latin America and Caribbean	4	11 406	9.6 (2.7–16.1)
Sub‐Saharan Africa	24	247 040	8.5 (4.2–12.7)
South Asia	13	166 149	2.7 (2.3–3.7)
East, Southeast Asia and Oceania	4	52 344	7.9 (6.7–8.8)
*By site*			
Urban	10	80 670	6.9 (4.2–14.3)
Rural	26	183 442	3.8 (2.7–7.4)
Mixed	9	212 827	11.2 (9.1–11.9)
*By period*			
Early (2000–2010)	12	85 052	4.9 (4.1–7.9)
Late (2011–2021)	33	391 887	5.9 (2.7–11.2)
*By GA measurement method*			
U/S	18	65 268	4.8 (2.7–7.9)
LMP	13	204 474	6.1 (3.2–11.9)
U/S and LMP	6	182 693	7.1 (4.5–9.6)
Not stated	8	24 504	6.8 (2.9–11.5)

Abbreviations: GA, gestational age; IQR, interquartile range; LMP, last menstrual period; U/S, ultrasound.

**TABLE 2:** Prevalence of large‐for‐gestational age by term‐preterm‐post‐term in 45 studies in low‐ and middle‐income countries.

Median prevalence	Total studies	Total live births	Preterm LGA	Term LGA	Post‐term LGA
Interquartile range	*k* = 45 studies	*n* = 476, 939	1.8 (0.7–4.6)	3.2 (0.9–5.1)	0.03 (0.00–0.21)
*By region*					
Latin America and Caribbean	4	11 406	0.6 (0.2–1.3)	7.3 (2.3–13.9)	0.23 (0.00–1.08)
Sub‐Saharan Africa	24	247 040	2.9 (0.7–6.9)	4.1 (2.2–5.3)	0.16 (0.00–0.25)
South Asia	13	166 149	1.7 (0.9–3.2)	0.7 (0.3–1.3)	0.00 (0.00–0.01)
East Asia, South‐east Asia and Oceania	4	52 344	2.2 (1.0–4.6)	5.5 (3.9–5.6)	0.11 (0.03–0.19)
*By site*					
Urban	10	80 670	0.8 (0.4–4.9)	4.0 (2.9–5.7)	0.00 (0.00–0.47)
Rural	26	183 442	1.7 (0.8–3.6)	1.4 (0.5–3.2)	0.01 (0.00–0.16)
Mixed	9	212 827	5.2 (2.6–7.8)	5.1 (4.1–6.6)	0.23 (0.12–0.32)
*By period*					
Early (2000–2010)	12	85 052	2.5 (1.6–3.9)	2.1 (0.4–5.2)	0.01 (0.00–0.30)
Late (2011–2021)	33	391 887	1.5 (0.6–5.2)	3.5 (1.6–5.1)	0.03 (0.00–0.17)
*By GA measurement method*					
U/S	18	65 268	0.9 (0.4–1.5)	3.2 (1.6–5.5)	0.00 (0.00–0.15)
LMP	13	204 474	3.6 (1.8–7.8)	1.7 (0.5–4.1)	0.03 (0.00–0.12)
U/S and LMP	6	182 693	4.0 (3.6–5.2)	2.9 (0.9–4.1)	0.08 (0.00–0.21)
Not stated	8	24 504	1.4 (0.4–2.5)	4.8 (2.1–6.2)	0.23 (0.08–0.42)

Abbreviations: GA, gestational age; LMP, last menstrual period; U/S, ultrasound.

LGA prevalence was lowest in rural settings (3.8%, IQR: 2.7%–7.4%) and highest in mixed rural and urban settings (11.2%, IQR: 9.1%–11.9%), followed by urban settings (6.9%, IQR: 4.2%–14.3%) (Table 1). We also found that studies conducted in rural areas showed a higher median prevalence of preterm LGAs at 1.7% (IQR: 0.8%–3.6%), whereas studies conducted in urban areas had a higher median prevalence of term LGAs (4.0%, IQR: 2.9%–5.7%) (Table 2).

Eighteen (out of 45) studies used U/S measurement methods, with 9 from sub‐Saharan Africa. Thirteen studies relied solely on LMP including 7 from South Asia and 5 from sub‐Saharan Africa. All studies using LMP alone showed higher LGA prevalence than those using U/S alone (Table S2). We found a median LGA prevalence of 4.8% (IQR: 2.7%–7.9%) in studies that used U/S, whereas median LGA prevalence was higher in studies incorporating LMP—(7.1%, IQR: 4.5%–9.6%) in those that used both U/S and LMP methods, and studies that used only the LMP method (6.1%, IQR: 3.2%–11.9%) (Table 1). Studies that used the LMP measurement method had a high median prevalence of preterm LGAs at 3.6% (IQR: 1.8–7.8), whereas U/S measurement had a high median prevalence of term LGAs at 3.2% (IQR: 1.6%–5.5%) (Table 2).

**FIGURE 1:**
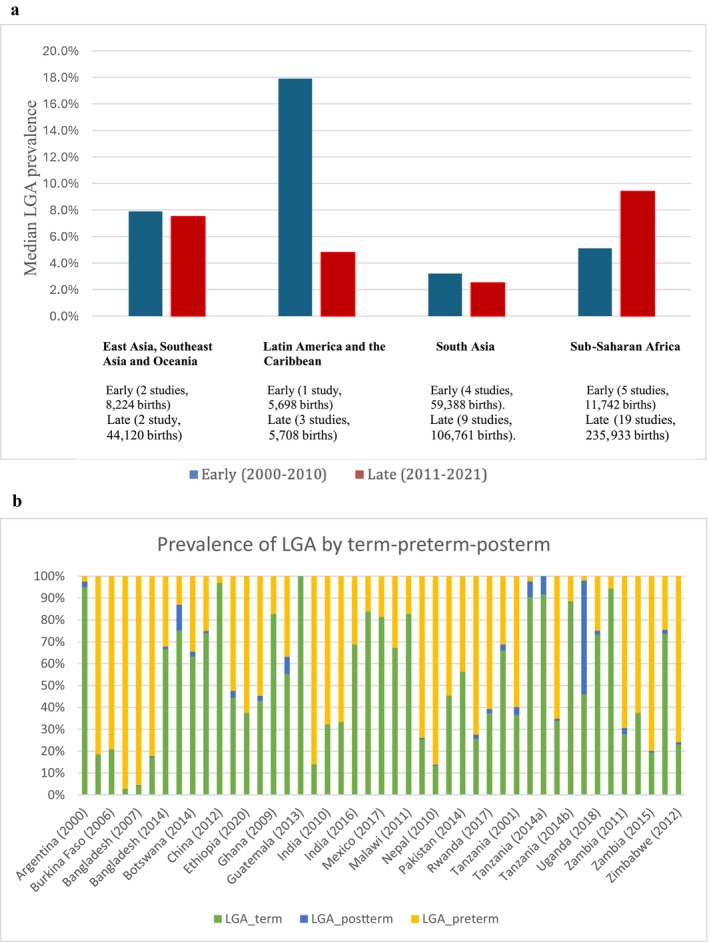
(a) Change in median prevalence of large‐for‐gestational‐age (LGA) infants by study period and region. (b) Prevalence of large‐for‐gestational age (LGA) by term–preterm–post‐term across all studies.

Furthermore, we observed temporal differences, with LGA prevalence increasing from a median of 4.9% (IQR: 4.1%–7.9%) between 2000 and 2010 to 5.9% (IQR: 2.7%–11.2%) between 2011 and 2021 (Table 1). The median prevalence of LGA in South Asia was 3.2% (4 studies, IQR: 2.5%–4.2%) between 2000 and 2010 and 2.5% (9 studies, IQR: 2.2%–3.7%) between 2011 and 2021. In Sub‐Saharan Africa, the median prevalence of LGA was 5.1% (5 studies, IQR: 4.8%–5.1%) between 2000 and 2010 and 9.4% (19 studies, IQR: 2.9%–13.4%) between 2011 and 2021 (Figure 1a). There were an inadequate number of studies to assess trends in Latin America and the Caribbean and East Asia. The median prevalence of post‐term LGA was low in all studies (Figure 1b).

Regarding macrosomia, we analysed 313 064 live births (25 studies) and identified 8411 cases. The overall median prevalence was 1.3% (IQR: 0.2%–2.4%). Most cases were term macrosomia (1.21%, IQR: 0.17–2.02), while preterm (0.04%, IQR: 0.00%–0.09%) and post‐term (0.08%, IQR: 0.00%–0.25%) macrosomia were lower (Table 3a). The median prevalence for birthweights ≥ 4500 g was 0.08% (IQR: 0.00%–0.26%), and for birthweights ≥ 5000 g, there were 110 babies. By region, the highest median prevalence of macrosomia was observed in Latin America and the Caribbean (3.1%, IQR: 0.7%–6.8%), while the lowest was in South Asia (0.1%, IQR: 0.0%–0.7%). Mixed sites had the highest prevalence (3.7%, IQR: 2.8%–4.4%), followed by urban (1.4%, IQR: 1.2%–5.0%) and rural areas (0.3%, IQR: 0.1%–1.3%). The median prevalence remained stable over time: 1.1% (IQR: 0.2%–3.1%) in older studies (2000–2010) and 1.3% (IQR: 0.5%–2.4%) in more recent studies (2011–2021) (Table 3).

**TABLE 3:** Prevalence of macrosomia in 25 studies in low‐ and middle‐income countries. (a) Prevalence by term, preterm and post‐term macrosomia. (b) Prevalence by grade of macrosomia.

(a)						
	Total studies	Total live births	Total macrosomia	Preterm macrosomia	Term macrosomia	Post‐term macrosomia
	*k* = 25	*n* = 313 064	*n* = 8411	*n* = 339	*n* = 6853	*n* = 1219
Median prevalence: (IQR)			1.3 (0.2–2.4)	0.04 (0.00–0.09)	1.21 (0.17–2.02)	0.08 (0.00–0.25)
*By region*						
Latin America and Caribbean	4	11 406	3.1 (07–6.8)	0.00 (0.00–0.02)	3.08 (0.70–6.45)	0.02 (0.00–0.34)
Sub‐Saharan Africa	10	209 228	1.9 (1.3–3.5)	0.09 (0.06–0.18)	1.63 (0.21–2.53)	0.19 (0.00–0.44)
South Asia	8	82 335	0.1 (0.0–0.7)	0.007 (0.0–0.05)	0.12 (0.02–0.51)	0.01 (0.00–0.07)
East Asia, South‐east Asia and Oceania	3	10 095	2.4 (1.3–3.8)	0.06 (0.02–0.10)	2.02 (1.01–3.49)	0.25 (0.21–0.36)
*By site*						
Urban	6	25 906	1.4 (1.2–5.0)	0.03 (0.04–0.09)	1.34 (1.18–4.93)	0.02 (0.00–0.19)
Rural	15	89 635	0.3 (0.1–1.8)	0.01 (0.00–0.09)	0.38 (0.04–1.54)	0.05 (0.00–0.19)
Mixed	4	197 523	3.7 (2.8–4.4)	0.12 (0.04–0.34)	2.68 (2.28–3.07)	0.79 (0.40–1.21)
*By period*						
Early (2000–2010)	8	42 735	1.1 (0.2–3.1)	0.01 (0.0–0.07)	1.14 (0.07–2.76)	0.11 (0.00–0.30)
Late (2011–2021)	17	270 329	1.3 (0.5–2.4)	0.06 (0.0–0.10)	1.21 (0.41–1.73)	0.08 (0.00–0.21)

(b)				
	Total studies	Total live births	≥ 4000 g	≥ 4500 g
	*k* = 25	*n* = 313 064	*n* = 8411	*n* = 1054
Median prevalence (IQR)			1.30 (0.28–2.43)	0.08 (0.00–0.26)
*By region*				
Latin America and Caribbean	4	11 406	3.13 (0.70–6.83)	0.24 (0.00–0.84)
Sub‐Saharan Africa	10	209 228	1.94 (1.30–3.35)	0.13 (0.00–0.30)
South Asia	8	82 335	0.19 (0.09–0.75)	0.02 (0.00–0.11)
East Asia, South‐east Asia and Oceania	3	10 095	2.41 (1.33–3.81)	0.11 (0.00–0.26)
*By site*				
Urban	6	25 906	1.48 (1.20–5.02)	0.27 (0.08–0.49)
Rural	15	89 635	0.38 (0.15–1.88)	0.00 (0.00–0.11)
Mixed	4	197 523	3.76 (2.88–4.48)	0.32 (0.22–0.56)
*By period*				
Early (2000–2010)	8	42 735	1.19 (0.21–3.11)	0.06 (0.00–0.21)
Late (2011–2021)	17	270 329	1.30 (0.58–2.43)	0.08 (0.00–0.30)

Abbreviations: GA, gestational age; IQR, interquartile range; LMP, last menstrual period; U/S, ultrasound.

## 4 Discussion

This analysis of sub‐national datasets that included 476 939 live births from 23 countries provides an updated view of LGA prevalence in LMICs. Additionally, we described the prevalence of macrosomia using data from 19 countries where individual birthweight data were available. Overall, the median prevalence of LGA across 45 studies was 5.1%, predominantly among term births, with the highest prevalence observed in Latin America and the Caribbean and the lowest in South Asia. This overall prevalence is lower than the expected 10% prevalence seen in optimally nourished populations using the INTERGROWTH‐21st standards. The prevalence of LGA was higher in studies that used LMP methods for estimating GA compared to studies using ultrasound alone and in those conducted in mixed urban–rural settings. We also observed a modest increase in LGA prevalence over time, rising from 4.9% in studies conducted between 2000 and 2010 to 5.9% in studies from 2011 to 2021. The median prevalence of macrosomia was 1.3%, primarily among term births, with lower prevalence observed in rural areas, and it remained relatively stable over time.

Studies using LMP estimates revealed a higher prevalence of preterm LGA cases, whereas studies dating pregnancies with more precise U/S measurements had a lower prevalence of preterm LGA babies. This can be attributed to the greater variance of GA when estimated by LMP dating compared to U/S measures. The differences in the reported LGA prevalence based on the GA estimation methods highlight the impact of measurement accuracy. Calculating GA by LMP is challenging due to recall bias and errors attributable to irregular menstrual cycles and is prone to random error with wider distribution at the tails and higher estimates of preterm birth [30, 31]. Conversely, U/S is generally more accurate, particularly in the first trimester where uncertainty in GA measurement is typically a few days. However, it may not fully account for normal variability in foetal growth patterns [30, 32]. Discrepancies between LMP and U/S methods affect regional comparisons, as reliance on LMP can lead to less precise gestational age estimates and misclassification of births, whereas regions using early ultrasound in urban or better‐resourced settings have more accurate GA dating and thus more reliable LGA or macrosomia prevalence estimates. It is well known that LMP tends to be more inaccurate, resulting in a wider distribution of GA (increasing the distribution of infants with low GA and overestimating the higher GA range), leading to higher preterm LGA rates compared to U/S, which provides more precise estimates [33]. However, the combined method moderates these effects, yielding intermediate prevalence rates [30, 34].

We found that LGA prevalence was highest in mixed urban–rural settings, which was unexpected and remains poorly explained. While differences in maternal nutrition, undiagnosed gestational diabetes and gestational age estimation methods may contribute, further studies are needed to clarify the underlying factors driving these regional disparities. The higher prevalence of LGA in urban compared to rural settings likely reflects a combination of factors including maternal overnutrition, metabolic dysregulation, environmental exposures, cultural influences, higher socioeconomic status, transitions to high‐calorie diets and improved healthcare access [35, 36, 37]. Preterm LGA was more prevalent in rural areas, possibly due to the existing socioeconomic disparities and limited access to early pregnancy U/S dating [38, 39, 40, 41]. In addition, several studies conducted in rural areas used the LMP method, which tends to overestimate prematurity [33, 42]. Variations in the median prevalence of LGA and macrosomia noted across studies within regions and across different time periods could be attributed to differences in the underlying data sources, populations and urban versus rural settings.

Across all studies, the median prevalence of LGA increased from 4.9% between 2000 and 2010 to 5.9% between 2011 and 2021. The observed increase in median LGA prevalence in sub‐Saharan Africa over the study period, compared to other regions, may reflect a combination of factors, including temporal changes in maternal BMI, clinical practices or data capture quality, and further analyses are needed to fully understand these dynamics. The lower prevalence of macrosomia in rural areas compared to urban and mixed settings highlights potential differences in maternal nutrition and may be attributed to higher rates of maternal undernutrition, which are often more prevalent in rural populations [43, 44]. This result also highlights the influence of lifestyle factors on the occurrence of large infants in various settings [45].

### 4.1 Interpretation

To date, LGA remains infrequently reported in LMICs compared to high‐income countries. Our analysis provides a description of the prevalence of LGA from sub‐national data in LMICs using the INTERGROWTH‐21st definition. The prevalence of LGA in participating LMICs appears to be lower than the expected 10% in a healthy population (as defined by the 90th centile with the INTERGROWTH‐21st—standard). The risks associated with LGA babies may be apparent from birth; however, research on the consequences of being born LGA in LMICs is limited. Knowing that the vulnerable newborn types classification offers a parsimonious approach to help identify determinants and examine the outcome of these infants, an important next step would be to assess these LGA groups (including LGA > 95th percentile) regarding neonatal mortality and health and survival beyond the neonatal period. In addition, future research should explore maternal and neonatal morbidity outcomes associated with macrosomia and LGA such as shoulder dystocia, birth trauma (e.g., fractures), prolonged neonatal admission, perineal injury, postpartum haemorrhage and potential neurodevelopmental sequelae. These outcomes are critical to guide patient‐centered counselling and inform shared decision‐making, particularly in the context of evolving clinical and legal standards such as the Montgomery ruling. While such considerations may not be universally applicable across all LMICs, integrating them into research agendas could help shape context‐appropriate clinical practice and policy.

Assessing large infants using weight for GA, as with LGA, considers birthweight, GA and sex, thereby providing a clearer view of growth relative to development and maturity at birth and facilitating targeted interventions [16]. However, this approach requires accurate GA data, which may not always be available or reliably measured. Using birthweight alone, as with macrosomia, is simpler and provides an immediate measure of high birth weight, which is useful for predicting delivery complications [16, 46]. However, it lacks the developmental context and may overlook the risks associated with GA. Although macrosomia is easier to apply universally and facilitates comparisons across populations and regions, it may oversimplify neonatal health by focusing solely on weight [16].

### 4.2 Strengths and Limitations

This individual participant data analysis included 476 939 live births from 23 countries and provides a large study size with a high level of completeness for the birth weight, GA and sex estimates, permitting a comprehensive assessment of the prevalence of LGA in different sub‐national populations. The use of the INTERGROWTH‐21st standards for GA and sex also enabled the first international comparisons and exploration of the prevalence of LGA infants in LMICs, although these standards may not fully capture population‐level diversity and could lead to over‐ or underestimation in specific settings.

Despite the high quality and completeness of the data, our study has some limitations. First, key variables related to noncommunicable diseases (NCDs) and maternal lifestyle factors were not available, limiting the depth of the conclusions drawn. Second, macrosomia could only be assessed in 25 of the 45 studies, restricting the robustness of findings related to this outcome. Third, the use of secondary data originally collected for other purposes—such as investigations of small‐for‐gestational‐age (SGA), infections or trials aiming to influence fetal growth—may introduce selection bias. In some cases, both control and intervention arms from randomised trials were included; while these studies generally enrolled unselected populations, this may have affected birth weight distributions. Moreover, the reliance on last menstrual period (LMP)—as opposed to more accurate gestational age dating methods like ultrasound—in most studies may have introduced measurement bias and potentially led to an overestimation of the proportion of preterm LGA infants. Nevertheless, this limitation also reflects the pragmatic nature of our study and its relevance to real‐world data contexts. Finally, the unequal representation of regions and the absence of several high‐burden countries—such as Nigeria, Indonesia, the Philippines, the Democratic Republic of the Congo and Kenya—limits the generalizability of our findings to all low‐ and middle‐income countries (LMICs).

Future research efforts should prioritise increasing the availability of antenatal ultrasound and the use of standardised ultrasound dating protocols to improve accuracy in estimating GA, thereby enhancing the reliability of LGA prevalence estimates. Incorporating these improvements can strengthen the validity and applicability of findings in future studies aiming to assess newborn health outcomes in LMICs.

## 5 Conclusion

This study provides the first comprehensive multi‐country analysis of LGA and macrosomia prevalence in LMICs. The prevalence of LGA (5.1%) is lower than that reported in HIC (20%); the highest prevalence was in Latin America and the Caribbean. There is limited data suggesting that LGA may be increasing in prevalence over the last decade.

To effectively address these challenges, strengthening health information systems and increasing access to early U/S for GA assessment in LMICs is imperative. The current reliance on aggregated cohort studies limits the accuracy and granularity of available data, hindering effective policymaking and intervention strategies. Enhanced data collection and surveillance capabilities will allow for a more nuanced understanding and precise monitoring of the prevalence of large vulnerable infants, their risk factors, and their associated health outcomes. Additionally, policies should prioritise integrating gestational diabetes screening and maternal nutrition programs into routine antenatal care to reduce the incidence of LGA and macrosomia. Implementing region‐specific strategies, such as maternal obesity prevention in Latin America and improved neonatal care in South Asia, will help mitigate LGA‐associated risks. Future research should focus on identifying maternal, nutritional and environmental factors contributing to regional variations in LGA and macrosomia prevalence.

In conclusion, there is a need for improved data collection and monitoring on the prevalence of large vulnerable infants in LMICs. By enhancing data infrastructure, we can better protect the health and well‐being of newborns at risk of complications related to excessive birth size or weight.

## Author Contributions

The Vulnerable Newborn Measurement Collaboration at Johns Hopkins University is led by E.A.H. and R.E.B. F.K.S. conceived the study and developed the analysis plan. All authors contributed to the study protocol and analysis approach. F.K.‐S., J.U., D.J.E. and E.A.H. carried out the descriptive analyses. The draft of the manuscript was prepared by F.K.‐S. with inputs of J.U., C.N., S.S., E.A.H., D.J.E., J.K., A.C.C.L. and R.E.B. All authors contributed to the revisions and approved the final manuscript.

## Conflicts of Interest

The authors declare no conflicts of interest.

## Supporting information


**Figure S1:** Flowchart of database construction for all studies (depicts how the pooled dataset was derived to calculate the prevalence of birth outcomes and vulnerable newborn types).

**Table S1:** Individual and median prevalence of large‐for‐gestational age in 45 studies in low‐ and middle‐income countries.

**Table S2:** Table of references for included studies.

## Data Availability

The data that support the findings of this study are available on request from the corresponding author. The data are not publicly available due to privacy or ethical restrictions.
