# Supplementary material for: Prevalence of Large‐for‐Gestational Age and Macrosomia Among Livebirths in 23 Low‐ and Middle‐Income Countries Between 2000 and 2021: An Individual Participant Data Analysis
Source: BJOG. 2025 Nov 10;132(Suppl 8):S97–S108. doi: 10.1111/1471-0528.70044 (PMC12678062; doi:10.1111/1471-0528.70044)
Supplement: Supplementary file 1 — Figure S1: Flowchart of database construction for all studies (depicts how the pooled dataset was derived to calculate the prevalence of birth outcomes and vulnerable newborn types). [file BJO-132-S97-s003.docx]

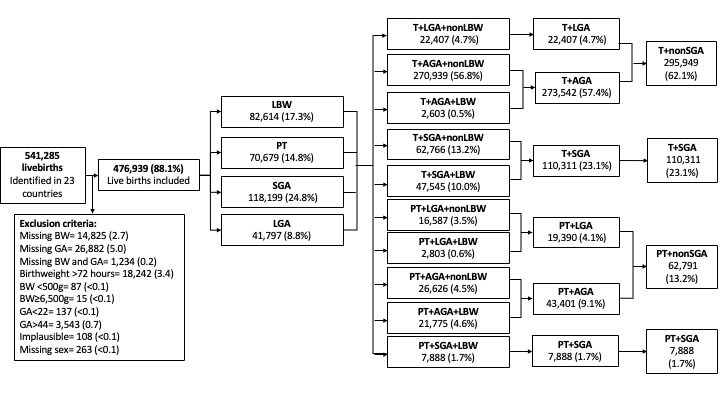


**Figure S1. Flowchart of database construction for all studies** (depicts how the pooled dataset was derived to calculate the prevalence of birth outcomes and vulnerable newborn types).
