# Supplementary material for: Prevalence of Large‐for‐Gestational Age and Macrosomia Among Livebirths in 23 Low‐ and Middle‐Income Countries Between 2000 and 2021: An Individual Participant Data Analysis
Source: BJOG. 2025 Nov 10;132(Suppl 8):S97–S108. doi: 10.1111/1471-0528.70044 (PMC12678062; doi:10.1111/1471-0528.70044)
Supplement: Supplementary file 2 — Table S1: Individual and median prevalence of large‐for‐gestational age in 45 studies in low‐ and middle‐income countries. [file BJO-132-S97-s001.docx]

**Table S1.** **Individual and median prevalence of large-for-gestational age in 45 studies in low- and middle-income countries**

| **Study** | **Total LGA N (%)** | | **Total livebirths** |
| --- | --- | --- | --- |
| **Latin America and Caribbean** |  | |  |
| Argentina (2000) | 1024 | (18.0) | 5698 |
| Brazil (2015) | 613 | (14.4) | 4257 |
| Guatemala (2013) | 4 | (0.7) | 565 |
| Mexico (2017) | 43 | (4.9 | 886 |
| **Median Prevalence (IQR)** | 11,406 | 9.6 (2.7-16.1) |  |
| **Sub-Saharan Africa** |  |  |  |
| Burkina Faso (2004) | 54 | (5.2) | 1045 |
| Burkina Faso (2006) | 48 | (4.6) | 1050 |
| Botswana (2014) | 18451 | (11.3) | 163928 |
| Ethiopia (2017) | 63 | (11.6 | 544 |
| Ethiopia (2020) | 8 | (1.4) | 556 |
| Ethiopia (2018) | 137 | (9.6) | 1424 |
| Ghana (2009) | 29 | (2.8) | 1037 |
| Ghana (2013) | 38 | (2.9) | 1291 |
| Malawi (2003) | 58 | (4.8) | 1199 |
| Malawi (2011) | 29 | (2.7 | 1074 |
| Rwanda (2017) | 490 | (17.7) | 2762 |
| Tanzania (2001) | 1044 | (13.7) | 7630 |
| Tanzania (2008) | 42 | (5.1) | 818 |
| Tanzania (2014a) | 12 | (2.9) | 407 |
| Tanzania (2010) | 997 | (12.0) | 8309 |
| Tanzania (2014b) | 312 | (13.5) | 2319 |
| Uganda (2016) | 25 | (3.9) | 635 |
| Uganda (2018) | 591 | (9.4) | 6255 |
| South Africa (2016) | 36 | (9.1) | 394 |
| Zambia (2011) | 5152 | (17.6) | 29207 |
| Zambia (2013) | 56 | (8.0) | 703 |
| Zambia (2015) | 2057 | (21.6) | 9509 |
| Zambia (2014) | 57 | (7.5) | 762 |
| Zimbabwe (2012) | 755 | (18.1) | 4182 |
| **Median Prevalence (IQR)** | 247,040 | 8.5 (4.2-12.7) |  |
| **South Asia** |  |  |  |
| Bangladesh Confidential data (2001) * | embargoed | | |
| Bangladesh Confidential data (2007) * | embargoed | | |
| Bangladesh, India, and Pakistan (2011-2014) | 1381 | (7.7) | 18007 |
| Bangladesh (2014) | 84 | (3.3) | 2572 |
| India (2000) | 114 | (2.8) | 4136 |
| India (2010) | 1130 | (2.5) | 44958 |
| India (2013) | 3 | (0.5) | 573 |
| India (2016) | 16 | (2.5) | 653 |
| Nepal (2010) | 894 | (3.8) | 23568 |
| Nepal (2002) | 499 | (2.3) | 21383 |
| Pakistan (2013) | 11 | (1.7) | 640 |
| Pakistan (2014) | 55 | (2.3) | 2415 |
| Sri Lanka (2015) | 563 | (4.2) | 13375 |
| **Median Prevalence (IQR)** | 166,149 | 2.7 (2.3-3.7) |  |
| **East, Southeast Asia and Oceania** |  |  |  |
| China (2002) | 327 | (7.5 | 4380 |
| China (2012) | 2511 | (5.9) | 42249 |
| Papua New Guinea (2009) | 171 | (9.1) | 1871 |
| Thailand (2000) | 327 | (8.5) | 3844 |
| **Median Prevalence (IQR)** | 52,344 | 7.9 (6.7-8.8) |  |

**Embargoed data**: Protected data that authors cannot expose at this time. **LGA**: large-for-gestational age
