# Supplementary material for: Prevalence of Large‐for‐Gestational Age and Macrosomia Among Livebirths in 23 Low‐ and Middle‐Income Countries Between 2000 and 2021: An Individual Participant Data Analysis
Source: BJOG. 2025 Nov 10;132(Suppl 8):S97–S108. doi: 10.1111/1471-0528.70044 (PMC12678062; doi:10.1111/1471-0528.70044)
Supplement: Supplementary file 3 — Table S2: Table of references for included studies. [file BJO-132-S97-s002.docx]

**Table S2**. **Table of references for included studies**

| Argentina (2000) | Grandi C, Del Pino M, Casale Aragon D, Dos Santos Rodrigues L, Cunha Cardoso V. Evaluation of the INTERGROWTH-21st project newborn standard for neonatal phenotypes and neonatal morbidity and mortality. Rev Fac Cien Med Univ Nac Cordoba. 2020 May 6;77(2):86-93. doi: 10.31053/1853.0605.v77.n2.28064. PMID: 32558510. |
| --- | --- |
| Bangladesh (2001) | Klemm RD, Merrill RD, Wu L, Shamim AA, Ali H, Labrique A, Christian P, West KP Jr. Low-birthweight rates higher among Bangladeshi neonates measured during active birth surveillance compared to national survey data. Matern Child Nutr. 2015 Oct;11(4):583-94. doi: 10.1111/mcn.12041. Epub 2013 May 6. PMID: 23647669; PMCID: PMC6860210. |
| Bangladesh (2007) | West KP Jr, Shamim AA, Mehra S, Labrique AB, Ali H, Shaikh S, Klemm RD, Wu LS, Mitra M, Haque R, Hanif AA, Massie AB, Merrill RD, Schulze KJ, Christian P. Effect of maternal multiple micronutrient vs iron-folic acid supplementation on infant mortality and adverse birth outcomes in rural Bangladesh: the JiVitA-3 randomized trial. JAMA. 2014 Dec 24-31;312(24):2649-58. doi: 10.1001/jama.2014.16819. PMID: 25536256. |
| Bangladesh, India, and Pakistan (2011-2014) | Saha SK, Schrag SJ, El Arifeen S, Mullany LC, Shahidul Islam M, Shang N, Qazi SA, Zaidi AKM, Bhutta ZA, Bose A, Panigrahi P, Soofi SB, Connor NE, Mitra DK, Isaac R, Winchell JM, Arvay ML, Islam M, Shafiq Y, Nisar I, Baloch B, Kabir F, Ali M, Diaz MH, Satpathy R, Nanda P, Padhi BK, Parida S, Hotwani A, Hasanuzzaman M, Ahmed S, Belal Hossain M, Ariff S, Ahmed I, Ibne Moin SM, Mahmud A, Waller JL, Rafiqullah I, Quaiyum MA, Begum N, Balaji V, Halen J, Nawshad Uddin Ahmed ASM, Weber MW, Hamer DH, Hibberd PL, Sadeq-Ur Rahman Q, Mogan VR, Hossain T, McGee L, Anandan S, Liu A, Panigrahi K, Abraham AM, Baqui AH. Causes and incidence of community-acquired serious infections among young children in south Asia (ANISA): an observational cohort study. Lancet. 2018 Jul 14;392(10142):145-159. doi: 10.1016/S0140-6736(18)31127-9. Epub 2018 Jul 6. PMID: 30025808; PMCID: PMC6053599. |
| Bangladesh (2014) | AMANHI (Alliance for Maternal and Newborn Health Improvement), Baqui A, Ahmed P, Dasgupta SK, Begum N, Rahman M, Islam N, Quaiyum M, Kirkwood B, Edmond K, Shannon C, Newton S, Hurt L, Jehan F, Nisar I, Hussain A, Nadeem N, Ilyas M, Zaidi A, Sazawal S, Deb S, Dutta A, Dhingra U, Ali SM, Hamer DH, Semrau KE, Straszak-Suri M, Grogan C, Bemba G, Lee AC, Wylie BJ, Manu A, Yoshida S, Bahl R. Development and validation of a simplified algorithm for neonatal gestational age assessment - protocol for the Alliance for Maternal Newborn Health Improvement (AMANHI) prospective cohort study. J Glob Health. 2017 Dec;7(2):021201. doi: 10.7189/jogh.07.021201. PMID: 29163937; PMCID: PMC5665676. |
| Botswana (2014) | Zash R, Holmes L, Diseko M, Jacobson DL, Brummel S, Mayondi G, Isaacson A, Davey S, Mabuta J, Mmalane M, Gaolathe T, Essex M, Lockman S, Makhema J, Shapiro RL. Neural-Tube Defects and Antiretroviral Treatment Regimens in Botswana. N Engl J Med. 2019 Aug 29;381(9):827-840. doi: 10.1056/NEJMoa1905230. Epub 2019 Jul 22. PMID: 31329379; PMCID: PMC6995896. |
| Brazil (2015) | Hallal PC, Bertoldi AD, Domingues MR, da Silveira MF, Demarco FF, da Silva ICM, Barros FC, Victora CG, Bassani DG. Cohort Profile: The 2015 Pelotas (Brazil) Birth Cohort Study. Int J Epidemiol. 2018 Aug 1;47(4):1048-1048h. doi: 10.1093/ije/dyx219. PMID: 29126133; PMCID: PMC6124621. |
| Burkina Faso (2004) | Roberfroid D, Huybregts L, Lanou H, Ouedraogo L, Henry MC, Meda N, Kolsteren P; MISAME study group. Impact of prenatal multiple micronutrients on survival and growth during infancy: a randomized controlled trial. Am J Clin Nutr. 2012 Apr;95(4):916-24. doi: 10.3945/ajcn.111.029033. Epub 2012 Feb 29. PMID: 22378724. |
| Burkina Faso (2006) | Huybregts L, Roberfroid D, Lanou H, Menten J, Meda N, Van Camp J, Kolsteren P. Prenatal food supplementation fortified with multiple micronutrients increases birth length: a randomized controlled trial in rural Burkina Faso. Am J Clin Nutr. 2009 Dec;90(6):1593-600. doi: 10.3945/ajcn.2009.28253. Epub 2009 Oct 7. PMID: 19812173. |
| China (2002) | Zeng L, Dibley MJ, Cheng Y, Dang S, Chang S, Kong L, Yan H. Impact of micronutrient supplementation during pregnancy on birth weight, duration of gestation, and perinatal mortality in rural western China: double blind cluster randomised controlled trial. BMJ. 2008 Nov 7;337:a2001. doi: 10.1136/bmj.a2001. Erratum in: BMJ. 2008;337:a2522. PMID: 18996930; PMCID: PMC2577799. |
| China (2012) | Qiu X, Lu JH, He JR, Lam KH, Shen SY, Guo Y, Kuang YS, Yuan MY, Qiu L, Chen NN, Lu MS, Li WD, Xing YF, Zhou FJ, Bartington S, Cheng KK, Xia HM. The Born in Guangzhou Cohort Study (BIGCS). Eur J Epidemiol. 2017 Apr;32(4):337-346. doi: 10.1007/s10654-017-0239-x. Epub 2017 Mar 20. PMID: 28321694. |
| Ethiopia (2017) | Tesfamariam K, Argaw A, Hanley-Cook GT, Gebreyesus SH, Kolsteren P, Belachew T, Van de Velde M, De Saeger S, De Boevre M, Lachat C. Multiple mycotoxin exposure during pregnancy and risks of adverse birth outcomes: a prospective cohort study in rural Ethiopia. Environ Int. 2022 Feb;160:107052. doi: 10.1016/j.envint.2021.107052. Epub 2021 Dec 21. PMID: 34952355. |
| Ethiopia (2018) | Chan GJ, Goddard FGB, Hunegnaw BM, Mohammed Y, Hunegnaw M, Haneuse S, Bekele C, Bekele D. Estimates of Stillbirths, Neonatal Mortality, and Medically Vulnerable Live Births in Amhara, Ethiopia. JAMA Netw Open. 2022 Jun 1;5(6):e2218534. doi: 10.1001/jamanetworkopen.2022.18534. PMID: 35749113; PMCID: PMC9233235. |
| Ethiopia (2020) | Lee AC, Abate FW, Mullany LC, Baye E, Berhane YY, Derebe MM, Eglovitch M, Fasil N, Olson IE, Kidane WT, Shiferaw T, Shiferie F, Tsegaye F, Tsegaye S, Yibeltal K, Chan GJ, Christian P, Isanaka S, Kang Y, Lu C, Mengistie MM, Molina RL, Stojanov MD, Van Dyk F, Tadesse AW, Wondale AT, Wylie BJ, Worku A, Berhane Y. Enhancing Nutrition and Antenatal Infection Treatment (ENAT) study: protocol of a pragmatic clinical effectiveness study to improve birth outcomes in Ethiopia. BMJ Paediatr Open. 2022 Jan;6(1):e001327. doi: 10.1136/bmjpo-2021-001327. PMID: 36053580; PMCID: PMC8762145. |
| Ghana (2009) | Adu-Afarwuah S, Lartey A, Okronipa H, Ashorn P, Zeilani M, Peerson JM, Arimond M, Vosti S, Dewey KG. Lipid-based nutrient supplement increases the birth size of infants of primiparous women in Ghana. Am J Clin Nutr. 2015 Apr;101(4):835-46. doi: 10.3945/ajcn.114.091546. Epub 2015 Feb 11. PMID: 25833980. |
| Ghana (2013) | Jack DW, Asante KP, Wylie BJ, Chillrud SN, Whyatt RM, Ae-Ngibise KA, Quinn AK, Yawson AK, Boamah EA, Agyei O, Mujtaba M, Kaali S, Kinney P, Owusu-Agyei S. Ghana randomized air pollution and health study (GRAPHS): study protocol for a randomized controlled trial. Trials. 2015 Sep 22;16:420. doi: 10.1186/s13063-015-0930-8. PMID: 26395578; PMCID: PMC4579662. |
| Guatemala (2013) | Hambidge KM, Krebs NF, Westcott JE, Garces A, Goudar SS, Kodkany BS, Pasha O, Tshefu A, Bose CL, Figueroa L, Goldenberg RL, Derman RJ, Friedman JE, Frank DN, McClure EM, Stolka K, Das A, Koso-Thomas M, Sundberg S; Preconception Trial Group. Preconception maternal nutrition: a multi-site randomized controlled trial. BMC Pregnancy Childbirth. 2014 Mar 20;14:111. doi: 10.1186/1471-2393-14-111. PMID: 24650219; PMCID: PMC4000057. |
| India (2000) | Rahmathullah L, Tielsch JM, Thulasiraj RD, Katz J, Coles C, Devi S, John R, Prakash K, Sadanand AV, Edwin N, Kamaraj C. Impact of supplementing newborn infants with vitamin A on early infant mortality: community based randomised trial in southern India. BMJ. 2003 Aug 2;327(7409):254. doi: 10.1136/bmj.327.7409.254. PMID: 12896935; PMCID: PMC167159. |
| India (2010) | Mazumder S, Taneja S, Bhatia K, Yoshida S, Kaur J, Dube B, Toteja GS, Bahl R, Fontaine O, Martines J, Bhandari N; Neovita India Study Group. Efficacy of early neonatal supplementation with vitamin A to reduce mortality in infancy in Haryana, India (Neovita): a randomised, double-blind, placebo-controlled trial. Lancet. 2015 Apr 4;385(9975):1333-42. doi: 10.1016/S0140-6736(14)60891-6. Epub 2014 Dec 11. PMID: 25499546. |
| India (2013) | Hambidge KM, Krebs NF, Westcott JE, Garces A, Goudar SS, Kodkany BS, Pasha O, Tshefu A, Bose CL, Figueroa L, Goldenberg RL, Derman RJ, Friedman JE, Frank DN, McClure EM, Stolka K, Das A, Koso-Thomas M, Sundberg S; Preconception Trial Group. Preconception maternal nutrition: a multi-site randomized controlled trial. BMC Pregnancy Childbirth. 2014 Mar 20;14:111. doi: 10.1186/1471-2393-14-111. PMID: 24650219; PMCID: PMC4000057. |
| India (2016) | Babu GR, Murthy GVS, Reddy Y, Deepa R, Yamuna A, Prafulla S, Krishnan A, Lobo E, Rathnaiah M, Kinra S. Small for gestational age babies and depressive symptoms of mothers during pregnancy: Results from a birth cohort in India. Wellcome Open Res. 2020 Feb 6;3:76. doi: 10.12688/wellcomeopenres.14618.3. PMID: 31828224; PMCID: PMC6892423. |
| Malawi (2003) | Luntamo M, Kulmala T, Mbewe B, Cheung YB, Maleta K, Ashorn P. Effect of repeated treatment of pregnant women with sulfadoxine-pyrimethamine and azithromycin on preterm delivery in Malawi: a randomized controlled trial. Am J Trop Med Hyg. 2010 Dec;83(6):1212-20. doi: 10.4269/ajtmh.2010.10-0264. PMID: 21118924; PMCID: PMC2990034. |
| Malawi (2011) | Ashorn P, Alho L, Ashorn U, Cheung YB, Dewey KG, Harjunmaa U, Lartey A, Nkhoma M, Phiri N, Phuka J, Vosti SA, Zeilani M, Maleta K. The impact of lipid-based nutrient supplement provision to pregnant women on newborn size in rural Malawi: a randomized controlled trial. Am J Clin Nutr. 2015 Feb;101(2):387-97. doi: 10.3945/ajcn.114.088617. Epub 2014 Dec 10. PMID: 25646337. |
| Mexico (2017) | Mendoza-Carrera CE, Acevedo-Gallegos S, Lumbreras-Márquez M, Gallardo-Gaona JM, Copado-Mendoza DY, Rodriguez-Sibaja MJ. Comparación de cuatro tablas de crecimiento fetal para la predicción de desenlaces perinatales adversos en un hospital de tercer nivel de México. Ginecol. obstet. Méx.  [revista en la Internet]. 2021;89( 9 ):704-714. Disponible en: http://www.scielo.org.mx/scielo.php?script=sci_arttext&pid=S0300-90412021000900704&lng=es.  Epub 04-Abr-2022.  <https://doi.org/10.24245/gom.v89i9.5817>. |
| Nepal (2002) | Mullany LC, Darmstadt GL, Khatry SK, Katz J, LeClerq SC, Shrestha S, Adhikari R, Tielsch JM. Topical applications of chlorhexidine to the umbilical cord for prevention of omphalitis and neonatal mortality in southern Nepal: a community-based, cluster-randomised trial. Lancet. 2006 Mar 18;367(9514):910-8. doi: 10.1016/S0140-6736(06)68381-5. PMID: 16546539; PMCID: PMC2367116. |
| Nepal (2010) | Katz J, SK Khatry, Shrestha L, Summers A, Visscher MO, Sherchand JB Tielsch JM, Subedi S, LeClerq SC, Mullany LC. Impact of topical applications of sunflower seed oil on neonatal mortality and morbidity in southern Nepal: a community-based, cluster-randomized trial. BMJ Global Health 2024;9:e013691 PMCID: PMC10910473 |
| Pakistan (2013) | Hambidge KM, Krebs NF, Westcott JE, Garces A, Goudar SS, Kodkany BS, Pasha O, Tshefu A, Bose CL, Figueroa L, Goldenberg RL, Derman RJ, Friedman JE, Frank DN, McClure EM, Stolka K, Das A, Koso-Thomas M, Sundberg S; Preconception Trial Group. Preconception maternal nutrition: a multi-site randomized controlled trial. BMC Pregnancy Childbirth. 2014 Mar 20;14:111. doi: 10.1186/1471-2393-14-111. PMID: 24650219; PMCID: PMC4000057. |
| Pakistan (2014) | AMANHI (Alliance for Maternal and Newborn Health Improvement), Baqui A, Ahmed P, Dasgupta SK, Begum N, Rahman M, Islam N, Quaiyum M, Kirkwood B, Edmond K, Shannon C, Newton S, Hurt L, Jehan F, Nisar I, Hussain A, Nadeem N, Ilyas M, Zaidi A, Sazawal S, Deb S, Dutta A, Dhingra U, Ali SM, Hamer DH, Semrau KE, Straszak-Suri M, Grogan C, Bemba G, Lee AC, Wylie BJ, Manu A, Yoshida S, Bahl R. Development and validation of a simplified algorithm for neonatal gestational age assessment - protocol for the Alliance for Maternal Newborn Health Improvement (AMANHI) prospective cohort study. J Glob Health. 2017 Dec;7(2):021201. doi: 10.7189/jogh.07.021201. PMID: 29163937; PMCID: PMC5665676. |
| Papua New Guinea (2009) | Unger HW, Ome-Kaius M, Wangnapi RA, Umbers AJ, Hanieh S, Suen CS, Robinson LJ, Rosanas-Urgell A, Wapling J, Lufele E, Kongs C, Samol P, Sui D, Singirok D, Bardaji A, Schofield L, Menendez C, Betuela I, Siba P, Mueller I, Rogerson SJ. Sulphadoxine-pyrimethamine plus azithromycin for the prevention of low birthweight in Papua New Guinea: a randomised controlled trial. BMC Med. 2015 Jan 16;13:9. doi: 10.1186/s12916-014-0258-3. PMID: 25591391; PMCID: PMC4305224. |
| Rwanda (2017) | Sayinzoga F, Lundeen T, Musange SF, Butrick E, Nzeyimana D, Murindahabi N, Azman-Firdaus H, Sloan NL, Benitez A, Phillips B, Ghosh R, Walker D. Assessing the impact of group antenatal care on gestational length in Rwanda: A cluster-randomized trial. PLoS One. 2021 Feb 2;16(2):e0246442. doi: 10.1371/journal.pone.0246442. PMID: 33529256; PMCID: PMC7853466. |
| South Africa (2016) | N/A |
| Sri Lanka (2015) | Senanayake H, Piccoli M, Valente EP, Businelli C, Mohamed R, Fernando R, Sakalasuriya A, Ihsan FR, Covi B, Wanzira H, Lazzerini M. Implementation of the WHO manual for Robson classification: an example from Sri Lanka using a local database for developing quality improvement recommendations. BMJ Open. 2019 Feb 19;9(2):e027317. doi: 10.1136/bmjopen-2018-027317. |
| Tanzania (2001) | Fawzi WW, Msamanga GI, Urassa W, Hertzmark E, Petraro P, Willett WC, Spiegelman D. Vitamins and perinatal outcomes among HIV-negative women in Tanzania. N Engl J Med. 2007 Apr 5;356(14):1423-31. doi: 10.1056/NEJMoa064868. PMID: 17409323. |
| Tanzania (2008) | Schmiegelow C, Minja D, Oesterholt M, Pehrson C, Suhrs HE, Boström S, Lemnge M, Magistrado P, Rasch V, Lusingu J, Theander TG, Bruun Nielsen B. Factors associated with and causes of perinatal mortality in northeastern Tanzania. Acta Obstet Gynecol Scand. 2012 Sep;91(9):1061-8. doi: 10.1111/j.1600-0412.2012.01478.x. PMID: 22676243. |
| Tanzania (2010) | Masanja H, Smith ER, Muhihi A, Briegleb C, Mshamu S, Ruben J, Noor RA, Khudyakov P, Yoshida S, Martines J, Bahl R, Fawzi WW; Neovita Tanzania Study Group. Effect of neonatal vitamin A supplementation on mortality in infants in Tanzania (Neovita): a randomised, double-blind, placebo-controlled trial. Lancet. 2015 Apr 4;385(9975):1324-32. doi: 10.1016/S0140-6736(14)61731-1. Epub 2014 Dec 11. PMID: 25499543; PMCID: PMC4419827. |
| Tanzania (2014a) | Hjort L, Lykke Møller S, Minja D, Msemo O, Nielsen BB, Lund Christensen D, Theander T, Nielsen K, Larsen LG, Grunnet LG, Groop L, Prasad R, Lusingu J, Schmiegelow C, Bygbjerg IC. FOETAL for NCD-FOetal Exposure and Epidemiological Transitions: the role of Anaemia in early Life for Non-Communicable Diseases in later life: a prospective preconception study in rural Tanzania. BMJ Open. 2019 May 22;9(5):e024861. doi: 10.1136/bmjopen-2018-024861. Erratum in: BMJ Open. 2019 Jun 28;9(6):e024861corr1. PMID: 31122967; PMCID: PMC6537995. |
| Tanzania (2014b) | AMANHI (Alliance for Maternal and Newborn Health Improvement), Baqui A, Ahmed P, Dasgupta SK, Begum N, Rahman M, Islam N, Quaiyum M, Kirkwood B, Edmond K, Shannon C, Newton S, Hurt L, Jehan F, Nisar I, Hussain A, Nadeem N, Ilyas M, Zaidi A, Sazawal S, Deb S, Dutta A, Dhingra U, Ali SM, Hamer DH, Semrau KE, Straszak-Suri M, Grogan C, Bemba G, Lee AC, Wylie BJ, Manu A, Yoshida S, Bahl R. Development and validation of a simplified algorithm for neonatal gestational age assessment - protocol for the Alliance for Maternal Newborn Health Improvement (AMANHI) prospective cohort study. J Glob Health. 2017 Dec;7(2):021201. doi: 10.7189/jogh.07.021201. PMID: 29163937; PMCID: PMC5665676. |
| Thailand (2000) | Isaranurug S, Mo-suwan L, Choprapawon C. A population-based cohort study of effect of maternal risk factors on low birthweight in Thailand. J Med Assoc Thai. 2007 Dec;90(12):2559-64. PMID: 18386704. |
| Uganda (2016) | Kajubi R, Ochieng T, Kakuru A, Jagannathan P, Nakalembe M, Ruel T, Opira B, Ochokoru H, Ategeka J, Nayebare P, Clark TD, Havlir DV, Kamya MR, Dorsey G. Monthly sulfadoxine-pyrimethamine versus dihydroartemisinin-piperaquine for intermittent preventive treatment of malaria in pregnancy: a double-blind, randomised, controlled, superiority trial. Lancet. 2019 Apr 6;393(10179):1428-1439. doi: 10.1016/S0140-6736(18)32224-4. Epub 2019 Mar 22. PMID: 30910321. |
| Uganda (2018) | Mulowooza J, Santos N, Isabirye N, Inhensiko I, Sloan NL, Shah S, Butrick E, Waiswa P, Walker D. Midwife-performed checklist and ultrasound to identify obstetric conditions at labour triage in Uganda: A quasi-experimental study. Midwifery. 2021 May;96:102949. doi: 10.1016/j.midw.2021.102949. Epub 2021 Feb 12. PMID: 33631411; PMCID: PMC7988503. |
| Zambia (2011) | Semrau KEA, Herlihy J, Grogan C, Musokotwane K, Yeboah-Antwi K, Mbewe R, Banda B, Mpamba C, Hamomba F, Pilingana P, Zulu A, Chanda-Kapata P, Biemba G, Thea DM, MacLeod WB, Simon JL, Hamer DH. Effectiveness of 4% chlorhexidine umbilical cord care on neonatal mortality in Southern Province, Zambia (ZamCAT): a cluster-randomised controlled trial. Lancet Glob Health. 2016 Nov;4(11):e827-e836. doi: 10.1016/S2214-109X(16)30215-7. Epub 2016 Sep 29. PMID: 27693439. |
| Zambia (2013) | Chaponda EB, Chico RM, Bruce J, Michelo C, Vwalika B, Mharakurwa S, Chaponda M, Chipeta J, Chandramohan D. Malarial infection and curable sexually transmitted and reproductive tract infections among pregnant women in a rural district of Zambia. Am J Trop Med Hyg. 2016;95(5):1069-1076. doi: 10.4269/ajtmh.16-0370. PMID: 27672205 PMCID: PMC5094219 |
| Zambia (2014) | AMANHI (Alliance for Maternal and Newborn Health Improvement), Baqui A, Ahmed P, Dasgupta SK, Begum N, Rahman M, Islam N, Quaiyum M, Kirkwood B, Edmond K, Shannon C, Newton S, Hurt L, Jehan F, Nisar I, Hussain A, Nadeem N, Ilyas M, Zaidi A, Sazawal S, Deb S, Dutta A, Dhingra U, Ali SM, Hamer DH, Semrau KE, Straszak-Suri M, Grogan C, Bemba G, Lee AC, Wylie BJ, Manu A, Yoshida S, Bahl R. Development and validation of a simplified algorithm for neonatal gestational age assessment - protocol for the Alliance for Maternal Newborn Health Improvement (AMANHI) prospective cohort study. J Glob Health. 2017 Dec;7(2):021201. doi: 10.7189/jogh.07.021201. PMID: 29163937; PMCID: PMC5665676. |
| Zambia (2015) | N/A |
| Zimbabwe (2012) | Humphrey JH, Mbuya MNN, Ntozini R, Moulton LH, Stoltzfus RJ, Tavengwa NV, Mutasa K, Majo F, Mutasa B, Mangwadu G, Chasokela CM, Chigumira A, Chasekwa B, Smith LE, Tielsch JM, Jones AD, Manges AR, Maluccio JA, Prendergast AJ; Sanitation Hygiene Infant Nutrition Efficacy (SHINE) Trial Team. Independent and combined effects of improved water, sanitation, and hygiene, and improved complementary feeding, on child stunting and anaemia in rural Zimbabwe: a cluster-randomised trial. Lancet Glob Health. 2019 Jan;7(1):e132-e147. doi: 10.1016/S2214-109X(18)30374-7. PMID: 30554749; PMCID: PMC6293965. |

* Some differences in reported birth counts reflect inclusion/exclusion criteria applied in the original studies versus the total live births provided for the pooled analysis
